# Risk factors for urinary retention after robot-assisted radical cystectomy with orthotopic neobladder diversion: a multicenter study

**DOI:** 10.1007/s11701-024-02099-y

**Published:** 2024-11-16

**Authors:** Younsoo Chung, Sangchul Lee, Byong Chang Jeong, Ja Hyeon Ku, Tae Gyun Kwon, Tae-Hwan Kim, Ji Youl Lee, Sung Hoo Hong, Woong Kyu Han, Won Sik Ham, Sung Gu Kang, Seok Ho Kang, Jong Jin Oh

**Affiliations:** 1 Department of Urology, Seoul National University Bundang Hospital, Seongnam, Korea; 2 Department of Urology, Samsung Medical Center, Sungkyunkwan University School of Medicine, Seoul, Korea; 3 Department of Urology, Seoul National University College of Medicine, Seoul, Korea; 4 Department of Urology, Kyungpook National University School of Medicine, Daegu, Korea; 5 Department of Urology, Seoul St. Mary’s Hospital, The Catholic University of Korea College of Medicine, Seoul, Korea; 6 Department of Urology, Severance Hospital, Yonsei University College of Medicine, Seoul, Korea; 7 Department of Urology, Korea University College of Medicine, Seoul, Korea; 8 Present Address: Building1 7th floor Urology office, 300-0, Bundang-Gu, Seongnam-si, Gyeonggi-Do Republic of Korea

**Keywords:** Bladder cancer, Body mass index, Female, Urination, Urination disorder

## Abstract

To determine risk factors for urinary retention (UR) after robot-assisted radical cystectomy (RALC) with orthotopic neobladder diversion. A total of 269 patients who underwent RALC with orthotopic neobladder diversion from 2008 to 2019 at seven tertiary hospitals were retrospectively analyzed. There were 68 patients who had UR (UR arm) and 201 patients who did not have UR (no-UR arm). UR was defined as voiding dysfunction without catheterization or more than 100 mL of residual urine after voiding. Preoperative demographics, perioperative factors, pathology outcomes, and postoperative complications of UR and no-UR arms were compared and predictors of UR were identified. Among demographic factors, only gender proportion showed a difference, with male proportion being significantly lower in the UR arm than in the no-UR arm (81% vs 92%, *p* = 0.010). For perioperative outcomes, anastomosis site stricture (27% vs 11%, *p* = 0.003) and length of hospital stays (23 days vs. 19 days, *p* = 0.001) were significantly higher in the UR arm than in the no-UR arm. In multiple logistic regression analysis, female (OR 3.32, 95% CI: 1.43–7.72) and body mass index (BMI) (OR 1.10, 95% CI 1.00–1.20) were UR predictors. UR after RALC with orthotopic neobladder diversion is significantly increased in females. Multiple logistic regression analysis identified female and BMI elevation as UR predictors.

## Introduction

Bladder cancer is diagnosed in approximately 573,000 individuals worldwide annually, ranking as the tenth most common cancer and causing around 213,000 deaths each year [1]. The current standard treatment for localized muscle invasive bladder cancer patients is neoadjuvant chemotherapy followed by radical cystectomy with lymph node dissection, and in very limited circumstances, trimodal therapy including radiation and chemotherapy can be considered [2]. There are three main types of urinary diversion: the ileal conduit, the neobladder, and the continent cutaneous diversion [3]. Of these, the neobladder is selected in 22% of cases due to its capacity to preserve the patient's body image by circumventing the necessity for a stoma, thereby enhancing patient satisfaction [4]. Since its introduction in 2003, there have been ongoing evaluations of the oncological and perioperative outcomes of robot-assisted laparoscopic cystectomy (RALC) [5–8]. Nevertheless, urinary retention (UR) following neobladder diversion remains a concern, with reported incidences ranging from 4 to 25% [9]. Urinary retention (UR) and voiding dysfunction can significantly impair patients' quality of life due to incomplete bladder emptying [10]. The aim of this multicenter retrospective study is to identify the risk factors associated with UR following RALC with neobladder diversion.

## Materials and methods

This study was approved by our Institutional Review Board (IRB) under IRB number B-2309–853-303. A multicenter retrospective study was conducted using RALC data from seven tertiary hospitals. Patients who underwent RALC with orthotopic neobladder diversion between years of 2009 and 2019 were included in this study. The recruitment period spanned from August 2009 to July 2019, with a mean follow-up duration of 35 months and a median follow-up duration of 24 months. The study continued until July 2019, or until the patient's death or loss to follow-up. UR was defined as the need for clean intermittent catheterization (CIC) or Foley catheter insertion due to inability to void voluntarily during the follow-up period or the presence of a post-void residual urine volume of 100 cc or greater during the follow-up period [11]. Inclusion criteria were patients who underwent RALC with orthotopic neobladder diversion. Exclusion criteria were patients who did not have a follow-up of their voiding function. Patients with UR were defined as the UR arm. Patients without UR was defined as the no-UR arm. The robotic surgical technique utilized the 4th arm and two assist ports (a 4 + 2 port configuration). Methods of urinary diversion included the Studer technique for extracorporeal diversion and the simple U technique for intracorporeal diversion [12, 13]. In male patients, a routine removal of the prostate was performed. The remaining urethra was treated in a similar manner to prostatectomy. The ureter was resected until a negative confirmation of cancer was observed in the frozen biopsy of the distal margin. However, if it was determined that there was not going to be enough length for an anastomosis to the neobladder, the resection was stopped. In the case of females, to avoid injury to external sphincter, the endopelvic fascia was left as intact as possible and the urethra was dissected at 1 cm or more distal to the neck of the bladder. The removal of the female reproductive organs was performed in cases where the tumor was located in the posterior region, involved the bladder neck, or was classified as T3 or higher. In females, maneuvers to prevent urinary retention included suspending the neobladder neck to the pubic bone and providing pelvic support by placing omentum in front of the uterus. Extracorporeal diversion was performed during the learning curve period, with no clear indication. Data collected included baseline characteristics such as age, gender, body mass index (BMI), American Society of Anesthesiologists (ASA) Classification, presence of diabetes mellitus (DM), hypertension (HTN), previous abdominal surgery, presence of neoadjuvant therapy, and follow-up duration. Perioperative data encompassed the intra/extracorporeal type, operative time, nerve saving status, Clavien grade of complications, length of postoperative hospital stay, and presence of anastomosis site strictures during follow-up. Anastomosis site stricture encompasses strictures occurring at both the ureter-neobladder anastomosis and the neobladder-urethra anastomosis. Logistic regression analysis was used to identify risk factors for UR. Age, gender, BMI, ASA classification, history of DM, corporeal method, and nerve saving procedure were subjected to logistic regression analyses. All tests were two-tailed and statistical significance was defined as p < 0.050. All missing proportion data were under 5% of each variable. All statistical analyses were performed using the Statistical Package for Social Sciences ver. 27 (IBM Corp., Armonk, NY, USA).

## Results

### Demographics

A total of 730 records were retrospectively reviewed. After applying exclusion criteria, a total of 269 patients were included in this study. Among them, 68 (25%) patients had UR (UR arm) while the remaining 201 (75%) patients did not have UR (no-UR arm). Baseline variables of patients in UR and no-UR groups were compared (Table 1). Only gender distribution showed a significant difference, with the proportion of females being higher in the UR arm than in the no-UR arm (19% vs 8%). Other baseline variables were comparable between the two groups.

**Table 1:** Demographics

Variable	UR	No-UR	p-value
Age, years, mean (SD)	61 (10)	60 (11)	0.667
Gender, *n* (%)			
Female	13 (19)	16 (8)	0.010
Male	55 (81)	185 (92)	
BMI, kg/m^2^, mean (SD)	25.0 (3.8)	24.3 (3.1)	0.196
ASA classification, *n* (%)			
1	29 (43)	76 (38)	0.793
2	37 (54)	118 (59)	
3	2 (3)	6 (3)	
HTN, *n* (%)	21 (31)	73 (36)	0.416
DM, *n* (%)	9 (13)	37 (18)	0.327
Previous Abdominal surgery, *n* (%)	11 (16)	46 (24)	0.227
Neoadjuvant therapy, *n* (%)	11 (16)	31 (15)	0.882
Follow-up months, mean (SD)	41 (37)	33 (30)	0.076

*UR* urinary retention, *SD* standard deviation, *BMI* body mass index, *ASA*, American Society of Anesthesiologists, *HTN* hypertension, *DM* diabetes mellitus

### Perioperative outcomes

Perioperative and postoperative outcomes are shown in Table 2. The distribution of corporeal type was comparable, with intracorporeal type accounting for 29% and 35% in UR and no-UR arms, respectively, and extracorporeal type accounting for 71% and 65% in UR and no-UR arms, respectively (*p* = 0.373). Nerve saving procedures were performed in 54% of both arms (*p* = 0.979). Clavien grade 3 or higher complications occurred in 24% of the UR arm and 21% of the no-UR arm (*p* = 0.648). However, strictures at the anastomotic site were significantly higher in the UR arm (27%) than in the no-UR arm (11%) (*p* = 0.003). The length of hospital stay was longer in the UR arm (23 days) than in the no-UR arm (19 days) (*p* = 0.008).

**Table 2:** Perioperative and postoperative outcomes

Variable	UR	No-UR	p-value
Intra/extra-corporeal method, *n* (%)			
Intracorporeal	20 (29)	71 (35)	0.373
Extracorporeal	48 (71)	130 (65)	
Operation time, min, mean (SD)	508 (146)	478 (133)	0.121
Nerve saving, n (%)	37 (54)	109 (54)	0.979
Hospital stay day, mean (SD)	23 (12)	19 (8)	0.008
Acute complication, n (%)	11 (16.2)	33 (16.4)	0.963
Urinary tract infection	4 (40)	13 (39)	
Urinary leakage	1 (10)	4 (12)	
Ileus/peritonitis	3 (30)	9 (27)	
Internal medical issue	2 (20)	7 (21)	
Clavien grade ≥ 3 complication, n (%)	16 (24)	42 (21)	0.648
Anastomosis site Stricture, n (%)	18 (26)	23 (11)	0.003
Ileal Urethra stricture	9 (13)	6 (3)	
Ileal Ureter stricture	9 (13)	14 (7)	
Combined	0 (0)	3 (2)	

*UR* urinary retention, *SD* standard deviation

### Predictors of developing urinary retention

Results of UR logistic regression analysis are shown in Table 3. In multi-variable logistic regression, female gender (OR 3.32, 95% CI 1.43–7.72) and higher BMI (OR 1.10, 95% CI: 1.00–1.20) were identified as significant factors of UR.

**Table 3:** Urinary retention odds ratio analyses

	*N*	Multi-variable	
		OR (95% CI)	*p*-value
Age	267	1.019 (0.991–1.048)	0.196
Gender			
Male	238	Ref	Ref
Female	29	3.324 (1.430–7.723)	0.005
BMI	267	1.096 (1.002–1.199)	0.044
ASA classification			
1	105	Ref	Ref
2	154	0.647 (0.336–1.245)	0.192
3	8	0.587 (0.096–3.591)	0.564
DM			
No	221	Ref	Ref
Yes	46	0.722 (0.307–1.697)	0.455
Intra/extra-corporeal method			
Intracorporeal method	91	Ref	Ref
Extracorporeal method	176	1.727 (0.848–3.519)	0.132
Nerve saving			
No	122	Ref	Ref
Yes	145	1.363 (0.722–2.570)	0.339

*OR* odds ratio, *CI* confidence interval, *BMI* body mass index, *ASA* American Society of Anesthesiologists, *DM* diabetes mellitus

## Discussion

In the UR arm, the proportion of females was higher, hospital stays were longer, and there were more anastomotic site strictures. Among these, ureteral anastomotic strictures may be related to retention, but were not considered a preoperative influencing factor, and because of the difficulty in differentiating them, were considered inappropriate for the study. There was no significant difference in the rate of complications during the hospital stay between the urinary retention (UR) and no-UR groups. The prolongation of the length of stay was due to transfers across multiple departments because of comorbidities, while in other cases, discharge occurred upon the completion of postoperative care. Also, the prolonged hospital stay in the UR group may be partly due to the increased need for repeated voiding trials, which were more frequently required to assess and manage postoperative bladder function. The longer hospital stay than other studies observed in our study is likely attributable to the distinctive healthcare insurance environment in Korea, which enables extended inpatient care due to lower hospital room charges. In terms of postoperative care, the removal of ureteral stents is typically conducted at the bedside between two and three days prior to discharge. The removal of the urethral catheter is performed before the patient is discharged, followed by a voiding trial. Additionally, a brief recovery program is conducted to provide verbal instructions to the patient. Compliance with the instructions is then monitored at follow-up visits.

The mechanism of UR after neobladder diversion has given rise to various hypotheses. These hypotheses can be broadly categorized into anatomical, neurogenic, and functional factors. There are common mechanisms that contribute to UR, although it is believed that they differ between genders. Both genders have common mechanisms, including mechanical factors such as formation of mucosal valves or strictures, which can be addressed through additional surgical interventions [14]. In addition to mechanical factors, the positioning of the neobladder during surgery also has an impact on UR. To prevent UR, it is essential to place the neobladder in the most dependent position and ensure effective funneling [9, 10, 15, 16]. In addition to factors discussed above, it is believed that UR after neobladder reconstruction in women is primarily influenced by anatomical and neurogenic factors, whereas functional factors are believed to play a role in men.

In women, numerous studies have identified the formation of the pouchcele as an anatomical factor in the development of voiding dysfunction. The female neobladder is often deficient in posterior support, which leads to the formation of a pouchcele that angulates the outlet of the neobladder and eventually results in obstruction [10, 17–21]. Precise removal of the neck of the bladder and the bladder outlet during operation is also very important. In men, the determination of the amount of urethra to be preserved is relatively straightforward due to presence of the prostate [19]. In women, however, this distinction is less clear, resulting in a tendency to preserve more of the bladder neck or bladder outlet than in men [19]. This can lead to the possibility of kinking, which may lead to a higher incidence of UR in women [19].

Mechanisms of neurogenic UR have shown inconsistent results in different studies. Attempts have been made to study the relationship between nerve-sparing procedures and the occurrence of UR, but evidence regarding neurogenic factor and UR is conflicting [14, 17, 18, 21–24]. Our study found no association between nerve-sparing and UR.

In men, in addition to common factors mentioned above, functional factor has been the most important contributor to UR [16]. Voiding after the formation of orthotopic neobladder requires a simultaneous increase in abdominal pressure and relaxation of the pelvic floor [20]. For patients who have difficulty with coordination, they are instructed to apply gentle manual pressure to the abdomen after relaxation of the pelvic floor in order to facilitate emptying of the bladder. Higher BMI might have an adverse effect on the control of intra-abdominal and pelvic floor pressures [25]. When operating on patients with a high BMI, it may be difficult to obtain deep pelvic access for dissection and challenging to perform cystectomy and orthotopic neobladder diversion [26]. In addition, patients with a higher BMI may have a shorter and thicker mesentery, making it difficult to achieve a water-tight anastomosis when the bowel is used for diversion [26]. Several studies have shown that BMI may increase the incidence of complications after cystectomy [25, 27, 28]. Our study confirmed that abnormal BMI was associated with an increase in urinary retention.

The strengths of this study are twofold. Firstly, unlike previous studies that obtained data from only one or two institutions, this study gathered data from seven different institutions, enhancing the robustness and generalizability of the findings. Secondly, while most studies have typically focused on female patients, this research includes results from both male and female patients, providing a more comprehensive understanding of anastomosis site strictures across genders. Despite these strengths, it was a retrospective and multicenter study, which might have introduced potential bias due to incomplete data and variable follow-up. As a multicenter study, heterogeneity among patient groups was inevitable. Minor differences in surgical techniques, approaches, and patient care could have influenced the study's outcomes. Additionally, voiding urodynamic studies and voiding cystourethrography were not used to evaluate voiding dysfunction. Over time, the incidence of urinary retention is expected to increase. However, our study only had two years of follow-up on average, which might have resulted in underestimation of future cases of urinary retention. In addition, the proportion of female patients who had urinary retention was relatively small, which might have led to an inaccurate assessment. A further limitation of our study is the lack of detailed data on the specific causes and management of urinary retention, including imaging findings and endoscopic procedures. Future research should aim to collect comprehensive data to better understand and manage urinary retention after robotic radical cystectomy with neobladder diversion.

## Conclusion

Urinary retention is one of the side effects associated with neobladder formation. In this study, the incidence of urinary retention was 25%, which required CIC or Foley catheter insertion. According to results of this study, urinary retention is more likely to occur in women and in patients with higher BMI.

## Data Availability

No datasets were generated or analysed during the current study.
